# Ketogenic Diet Enhances the Cholesterol Accumulation in Liver and Augments the Severity of CCl_4_ and TAA-Induced Liver Fibrosis in Mice

**DOI:** 10.3390/ijms22062934

**Published:** 2021-03-13

**Authors:** Yi-Jen Liao, Yuan-Hsi Wang, Chien-Ying Wu, Fang-Yu Hsu, Chia-Ying Chien, Yi-Chieh Lee

**Affiliations:** School of Medical Laboratory Science and Biotechnology, College of Medical Science and Technology, Taipei Medical University, Taipei 110, Taiwan; m609105001@tmu.edu.tw (Y.-H.W.); b614107065@tmu.edu.tw (C.-Y.W.); b507106042@tmu.edu.tw (F.-Y.H.); m609108003@tmu.edu.tw (C.-Y.C.); b614106056@tmu.edu.tw (Y.-C.L.)

**Keywords:** high-fat ketogenic diet, liver fibrosis, β-hydroxybutyrate, acetoacetate, hepatic stellate cells

## Abstract

Persistent chronic liver diseases increase the scar formation and extracellular matrix accumulation that further progress to liver fibrosis and cirrhosis. Nevertheless, there is no antifibrotic therapy to date. The ketogenic diet is composed of high fat, moderate to low-protein, and very low carbohydrate content. It is mainly used in epilepsy and Alzheimer’s disease. However, the effects of the ketogenic diet on liver fibrosis remains unknown. Through ketogenic diet consumption, β-hydroxybutyrate (bHB) and acetoacetate (AcAc) are two ketone bodies that are mainly produced in the liver. It is reported that bHB and AcAc treatment decreases cancer cell proliferation and promotes apoptosis. However, the influence of bHB and AcAc in hepatic stellate cell (HSC) activation and liver fibrosis are still unclear. Therefore, this study aimed to investigate the effect of the ketogenic diet and ketone bodies in affecting liver fibrosis progression. Our study revealed that feeding a high-fat ketogenic diet increased cholesterol accumulation in the liver, which further enhanced the carbon tetrachloride (CCl_4_)- and thioacetamide (TAA)-induced liver fibrosis. In addition, more severe liver inflammation and the loss of hepatic antioxidant and detoxification ability were also found in ketogenic diet-fed fibrotic mouse groups. However, the treatment with ketone bodies (bHB and AcAc) did not suppress transforming growth factor-β (TGF-β)-induced HSC activation, platelet-derived growth factor (PDGF)-BB-triggered proliferation, and the severity of CCl_4_-induced liver fibrosis in mice. In conclusion, our study demonstrated that feeding a high-fat ketogenic diet may trigger severe steatohepatitis and thereby promote liver fibrosis progression. Since a different ketogenic diet composition may exert different metabolic effects, more evidence is necessary to clarify the effects of a ketogenic diet on disease treatment.

## 1. Introduction

Liver fibrosis is caused by a protracted wound healing process that ultimately results in liver failure [1]. Chronic liver diseases (caused by the hepatitis virus infection, alcohol abuse, non-alcoholic fatty liver disease (NAFLD)/non-alcoholic steatohepatitis (NASH), etc.) can progress to advanced fibrosis [2]. The terminal stage of progressive liver fibrosis is cirrhosis and it has a 1–7% risk of developing primary hepatocellular carcinoma yearly [1]. It is reported that cirrhosis affects about 1–2% of the global population and leads to more than 1 million deaths annually worldwide [3]. However, no antifibrotic therapy has been approved to date.

Hepatic stellate cells (HSCs) are considered as a central driver of liver fibrosis [4]. HSCs maintain a non-proliferative quiescent phenotype in the normal liver. While under liver injury, HSCs become activated and transdifferentiated from vitamin-A-storing cells to myofibroblasts [5]. The activated HSCs are characterized by enhanced extracellular matrix (ECM) production and are proliferative, chemotactic, contractile, and inflammatory [5]. Previous studies have proposed several mechanisms that regulate HSC activation including fibrogenic and proliferative cytokines, HSC–ECM interactions, and the hedgehog pathway [4]. Especially, transforming growth factor-β (TGF-β) and platelet-derived growth factor (PDGF) are generally considered the most important cytokines in regulating HSC activation [4]. TGF-β is a fibrogenic cytokine that induces downstream SMAD protein phosphorylation and promotes α-smooth muscle actin (α-SMA) and collagen production by activated HSCs [6,7]. PDGF is a chemoattractant that drives HSC proliferation and migration via MAPK and AKT pathway activation [8]. Therefore, targeting regulatory mechanisms will provide advantages in developing antifibrotic strategies.

Currently, the ketogenic diet has emerged as a potential therapy for different diseases. The ketogenic diet is composed of high fat, moderate to low-protein, and very low carbohydrate content [9]. It is reported that ketogenic diets are highly appreciated in the therapy of epileptic seizures and reduces 50% of the frequency of seizures after using it for 3 months [10]. In liver disease, the ketogenic diet ameliorated NAFLD progression by promoting weight loss and decreasing the amount of triglyceride in the liver [11]. Our lab previously reported that a ketogenic diet significantly inhibited liver cancer cell growth in mice [12,13]. Conversely, it is also reported that the ketogenic diet aggravates neurodegeneration and mitochondrial deterioration in a transgenic mouse model [14]. However, the influence of the ketogenic diet on liver fibrosis is still unknown.

While consuming a ketogenic diet, the increased fat and limited carbohydrate metabolism lead to increased ketone body production in the body, which is also called physiologic ketosis [9]. Notably, the production of ketone bodies from fatty acids primarily occurs in the liver and is further transported to the extrahepatic tissues [15]. The three endogenous ketone bodies include β-hydroxybutyrate (bHB), acetoacetate (AcAc), and acetone [15]. In particular, bHB and AcAc are the two predominant ketone bodies in the human body [16]. It is reported that treatment with bHB inhibited lipopolysaccharide-induced inflammatory genes expression in murine microglial BV-2 cells [17]. Besides, previous studies indicated that both bHB and AcAc treatment decreased cell growth and increased cell apoptosis in pancreatic cancer cells [18]. However, the role of ketone bodies in affecting HSC activation and liver fibrosis remains unknown. Therefore, the aim of this study was to investigate the effect of the ketogenic diet and ketone bodies in affecting liver fibrosis progression.

## 2. Results

### 2.1. Ketogenic Diet Enhanced Carbon Tetrachloride-Induced Liver Fibrosis

**Figure 1:**
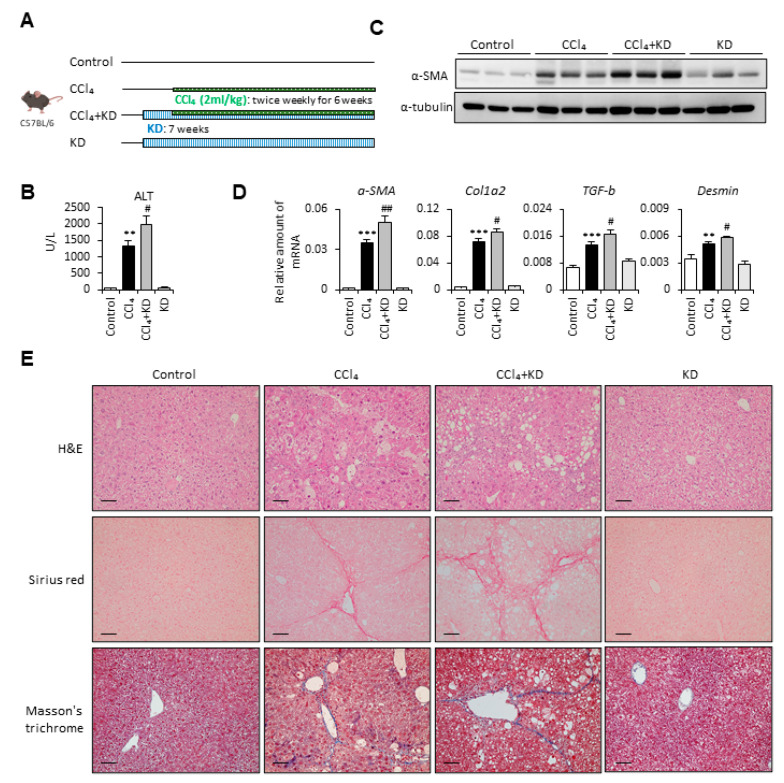
Ketogenic diet enhanced the severity of carbon tetrachloride (CCl_4_)-induced liver fibrosis. (**A**) Study design of the in vivo experiment. (**B**) Serum samples were collected at the end of the experiment, and the serum alanine aminotransferase (ALT) was assessed. (**C**) Representative results from Western blot analyses of α-SMA expression and (**D**) qPCR analyses of α-SMA, Col1a2, transforming growth factor-β (TGF-β), and Desmin gene expression. (**E**) Representative hematoxylin and eosin (H&E), Sirius red, and Masson’s trichrome staining images of liver tissues. Scale bars: 0.1 mm. **, *p* < 0.01; ***, *p* < 0.001 vs. white bar. #, *p* < 0.05; ##, *p* < 0.01 vs. black bar. *N* = 5 in each group. Data are shown as means ± SEM.

To define the role of the ketogenic diet in affecting liver fibrosis progression, we first used the carbon tetrachloride (CCl_4_)-induced liver fibrosis mouse model in our experiment (Figure 1A). The results demonstrated that the ketogenic diet-fed fibrotic mouse group had markedly increased serum alanine aminotransferase (ALT) levels compared with the CCl_4_-alone group (Figure 1B). Furthermore, we examined the protein and mRNA expression levels of fibrotic markers. In Figure 1C, we show that mice fed with a ketogenic diet significantly increased CCl_4_-induced α-SMA expression. The mRNA expression levels of α-SMA, collagen type 1 alpha 2 (Col1a2), TGF-β, and Desmin were also increased in the ketogenic diet-fed fibrotic mouse group compared with the CCl_4_-alone group (Figure 1D). Besides, the results from hematoxylin and eosin (H&E) staining indicated that ketogenic diet supplementation enhanced CCl_4_-induced liver damage (Figure 1E). The Sirius red staining and Masson’s trichrome staining were also performed to evaluate the collagen deposition in the liver from four groups. We showed that the collagen deposition was markedly increased in the liver from the ketogenic diet-fed fibrotic mouse group (Figure 1E). Notably, supplementation with the ketogenic diet alone did not induce liver fibrosis or cause any liver damage.

### 2.2. Ketogenic Diet Increased the Severity of Thioacetamide-Induced Liver Fibrosis

**Figure 2:**
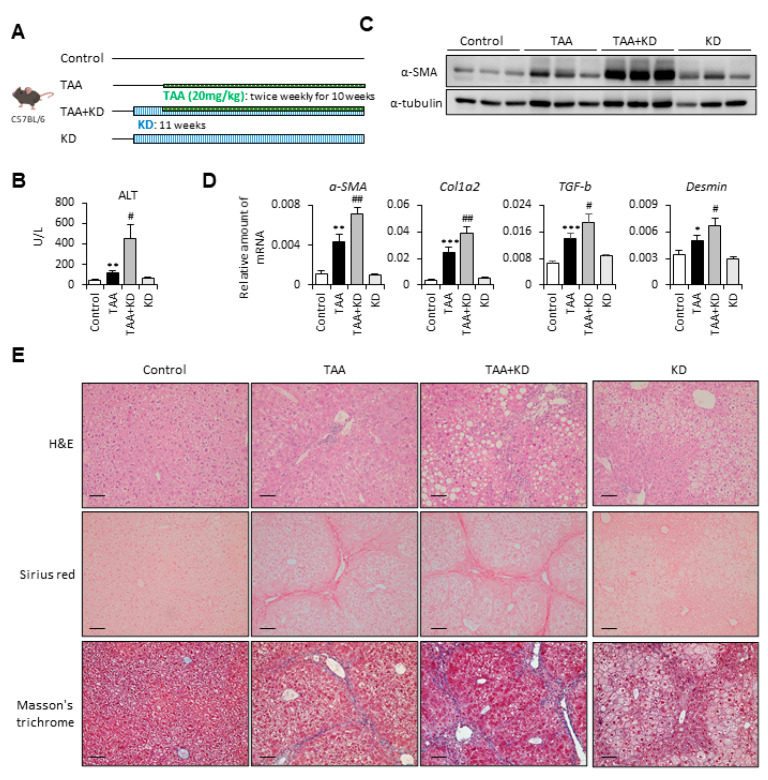
Ketogenic diet enhanced the severity of thioacetamide (TAA)-induced liver fibrosis. (**A**) Study design of the in vivo experiment. (**B**) Serum samples were collected at the end of the experiment, and the serum ALT was assessed. (**C**) Representative results from Western blot analyses of α-SMA expression and (**D**) qPCR analyses of α-SMA, Col1a2, TGF-β, and Desmin gene expression. (**E**) Representative H&E, Sirius red, and Masson’s trichrome staining images of liver tissues. Scale bars: 0.1 mm. *, *p* < 0.05; **, *p* < 0.01; ***, *p* < 0.001 vs. white bar. #, *p* < 0.05; ##, *p* < 0.01 vs. black bar. *N* = 5 in each group. Data are shown as means ± SEM.

To confirm our findings, we used thioacetamide (TAA) to set up the second fibrotic mouse model (Figure 2A). In Figure 2B, we show that the ketogenic diet-supplemented fibrotic mouse group had markedly increased serum ALT levels compared with the TAA-alone group. Besides, mice fed with a ketogenic diet significantly increased TAA-induced protein and mRNA expression levels of fibrotic markers, including α-SMA, Col1a2, TGF-β, and Desmin (Figure 2C,D). Moreover, the results of histologic examination revealed that the ketogenic diet significantly increased TAA-induced liver damage and collagen deposition (Figure 2E). These data demonstrate that the ketogenic diet increased the severity of both CCl_4_- and TAA-induced liver fibrosis.

### 2.3. Treatment with bHB Did Not Influence TGF-β-Induced HSC Cell Activation

**Figure 3:**
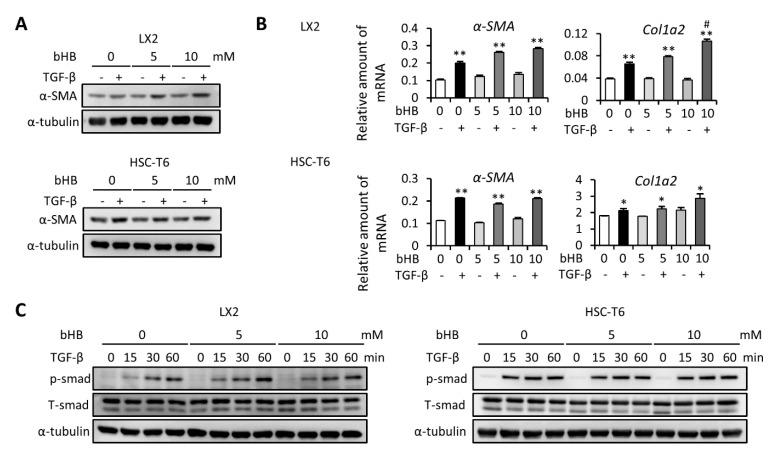
Effects of β-hydroxybutyrate (bHB) on TGF-β1-induced LX2 and HSC-T6 cell activation. (**A**,**B**) LX2 and HSC-T6 cells were treated with 10 ng/mL TGF-β and 0, 5, or 10 mM of bHB for 24 h. Western blot and qPCR were used to evaluate the protein and mRNA expression of α-SMA and Col1a2. (**C**) LX2 and HSC-T6 cells were treated with 0, 5, or 10 mM of bHB for 24 h and then subjected to 10 ng/mL TGF-β for the indicated time periods, and the lysates were analyzed by Western blot and quantification to detect SMAD protein phosphorylation. *, *p* < 0.05; **, *p* < 0.01 vs. relative control. #, *p* < 0.05 vs. black bar. Data are shown as means ± SD.

To test whether the level of ketone bodies is a major determinant of enhancing the severity of liver fibrosis, we first evaluated the effect of bHB on HSC activation. We found that treatment with bHB increased the TGF-β-induced α-SMA expression in LX2 cells, while there was no significant effect in HSC-T6 cells (Figure 3A). Besides, the mRNA expression level of α-SMA was not changed after treatment with bHB in both cells (Figure 3B). The mRNA expression levels of Col1a2 were similar after treatment with 5 mM of bHB in both cells but slightly increased after treatment with 10 mM of bHB in LX2 cells (Figure 3B). We also evaluated the TGF-β-induced phosphorylation of SMAD protein. The results showed that after treatment with 5 or 10 mM of bHB, the protein expression level of phosphor-SMAD were not significant changed in both LX2 and HSC-T6 cells (Figure 3C). Taken together, these results suggest that bHB is not able to affect TGF-β-induced HSC cell activation, which means that bHB is not the key factor for the effect of increasing the severity of liver fibrosis.

### 2.4. AcAc Treatment Shows No Effect on TGF-β-Induced HSC Cell Activation

**Figure 4:**
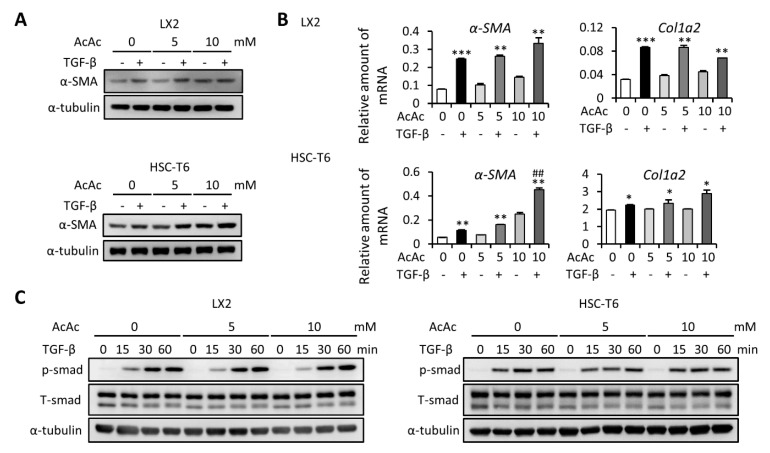
Effects of acetoacetate (AcAc) on TGF-β1-induced LX2 and HSC-T6 cell activation. (**A**,**B**) LX2 and HSC-T6 cells were treated with 10 ng/mL TGF-β and 0, 5, or 10 mM of AcAc for 24 h. Western blot and qPCR were used to evaluate the protein and mRNA expression of α-SMA and Col1a2. (**C**) LX2 and HSC-T6 cells were treated with 0, 5, or 10 mM of AcAc for 24 h and then subjected to 10 ng/mL TGF-β for the indicated time periods, and the lysates were analyzed by Western blot and quantification to detect SMAD protein phosphorylation. *, *p* < 0.05; **, *p* < 0.01; ***, *p* < 0.001 vs. relative control. ##, *p* < 0.01 vs. black bar. Data are shown as means ± SD.

Since AcAc is another important type of ketone body, we next evaluated the effect of AcAc on HSC activation. We found that treatment with AcAc did not influence TGF-β-induced protein and mRNA expression levels of α-SMA, and Col1a2 expression in LX2 cells (Figure 4A,B upper). Conversely, treatment with AcAc increased the protein and mRNA expression levels of α-SMA in HSC-T6 cells, while the mRNA expression level of Col1a2 was not changed (Figure 4A,B lower). Furthermore, treatment with AcAc did not influence TGF-β-induced phosphorylation of SMAD protein in both LX2 and HSC-T6 cells. These data indicated that AcAc is also not able to affect TGF-β-induced HSC cell activation. It is well known that PDGF-mediated HSC proliferation is also an important aspect of the progression of liver fibrosis [19]. Therefore, we further analyzed the effect of both bHB and AcAc on PDGF-induced mitogen-activated protein kinases (MAPK) and AKT signaling activation in HSC cells. Our results demonstrated that treatment with either bHB or AcAc did not influence the PDGF-induced phosphorylation of mitogen-activated protein kinase kinase (MEK), extracellular signal-regulated kinases (ERK), P38, JNK, and AKT in both LX2 and HSC-T6 cells (Supplementary Figure S1A,B). In addition, the cell viability showed no difference among 0, 1, 5, and 10 mM of bHB and AcAc treatment of HSC cells (Supplementary Figure S1C). These data indicated that both bHB and AcAc did not influence HSC proliferation and PDGF-induced MAPK and AKT signaling.

### 2.5. Administration of bHB and AcAc Did Not Ameliorate CCl_4_-Induced Liver Fibrosis in Mice

**Figure 5:**
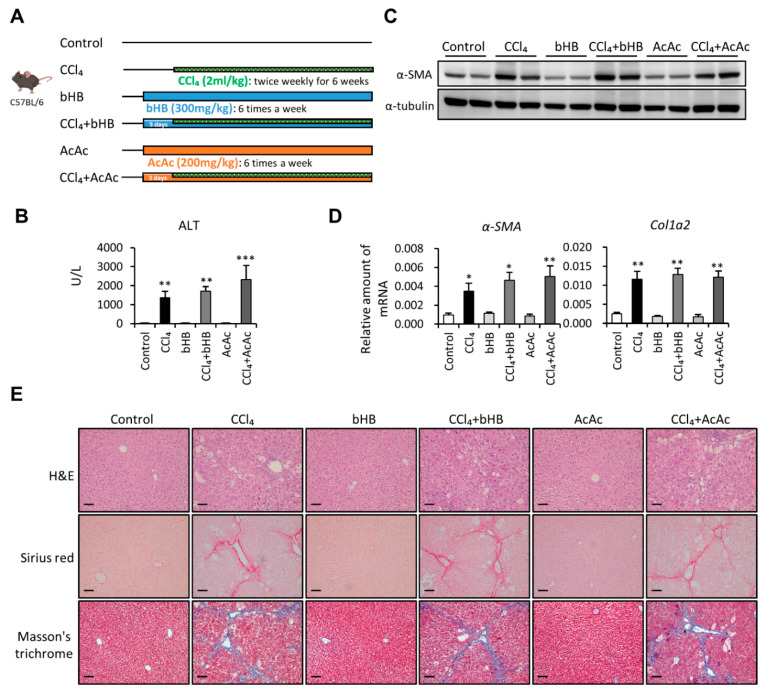
Ketone body supplementation did not influence the severity of CCl_4_-induced liver fibrosis. (**A**) Study design of the in vivo experiment. (**B**) Serum samples were collected at the end of the experiment, and the serum ALT was assessed. (**C**) Representative results from Western blot analyses of α-SMA expression and (**D**) qPCR analyses of α-SMA and Col1a2 gene expression. (**E**) Representative H&E, Sirius red, and Masson’s trichrome staining images of liver tissues. Scale bars: 0.1 mm. *, *p* < 0.05; **, *p* < 0.01; ***, *p* < 0.001 vs. relative control. *N* = 5 in each group. Data are shown as mean ± SEM.

Next, we used the CCl_4_-induced liver fibrosis mouse model with intraperitoneal injection of both bHB and AcAc to further confirm what we had observed in the cell experiments (Figure 5A). The results showed that CCl_4_ successfully induced liver fibrosis with increased serum ALT levels, while treatment with either bHB or AcAc did not influence the results (Figure 5B). In addition, ketone body supplementation with either bHB or AcAc did not influence CCl_4_—induced α-SMA and Col1a2 expression in the liver (Figure 5C,D). Moreover, the results of histologic examination revealed that bHB or AcAc supplementation did not affect CCl_4_-induced liver damage and collagen deposition (Figure 5E). These data demonstrated that intraperitoneal injection of both bHB and AcAc did not affect the severity of CCl_4_-induced liver fibrosis.

### 2.6. Ketogenic Diet Increased the Cholesterol Quantity, Which Further Enhanced Liver Inflammation and Reduced Antioxidant and Detoxification Gene Expression

**Figure 6:**
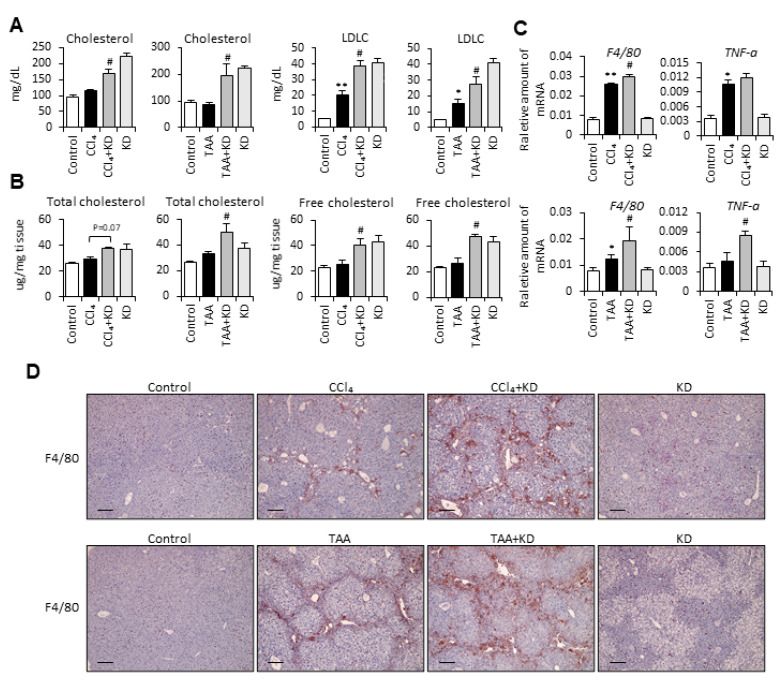

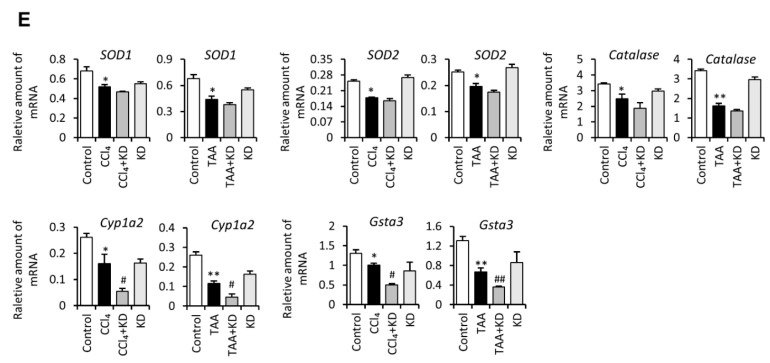
Ketogenic diet increased the cholesterol quantity and further enhanced CCl_4_- and TAA- induced liver fibrosis, liver inflammation, and the loss of antioxidant and detoxification ability. (**A**) Serum samples were collected at the end of the experiment, and the serum cholesterol and low-density lipoprotein cholesterol (LDLC) were assessed. (**B**) Intracellular total and free cholesterol were measured by using a colorimetric cholesterol quantification kit. (**C**) QPCR analyses of F4/80 and TNF-α gene expression. (**D**) Representative F4/80 immunohistochemistry staining of livers from each treatment group. Scale bars: 0.1 mm. (**E**) QPCR analyses of SOD1, SOD2, Catalase, Cyp1a2, and Gsta3 gene expression. *, *p* < 0.05; **, *p* < 0.01 vs. white bar. #, *p* < 0.05; ##, *p* < 0.01 vs. black bar. *N* = 5 in each group. Data are shown as means ± SEM.

From Figure 3, Figure 4 and Figure 5, we concluded that ketogenic diet-induced ketone body production in mice is not the key factor that augments the severity of CCl_4_- and TAA-induced liver fibrosis in mice. Surprisingly, when we examined the result of H&E staining again, we found that the ketogenic diet-supplemented fibrotic mouse group possessed more lipid accumulation in the liver tissue (Figure 1E and Figure 2E). Previous studies reported that higher cholesterol levels in the liver increased the severity of liver fibrosis [20]. Since the ketogenic diet is composed of a high percentage of fat, it is worth studying whether mice fed with a ketogenic diet affected the cholesterol composition. Therefore, we first examined the serum level of cholesterol and low-density lipoprotein cholesterol (LDLC) in different groups of mice. The results showed that both cholesterol and LDLC quantity in serum were higher in the ketogenic diet-fed fibrotic mouse group compared with the CCl_4_- or TAA-alone groups (Figure 6A). The quantification of total and free cholesterol in the liver also demonstrated that a ketogenic diet increased the amount of cholesterol in the liver (Figure 6B). Next, we examined whether the increased cholesterol amount further influenced liver inflammation or antioxidant and detoxification ability. The results showed that the mRNA and protein expression levels of TNF-α and F4/80 were elevated in the ketogenic diet-fed fibrotic mouse group compared with the CCl_4_- or TAA-alone groups (Figure 6C,D). The mRNA expression levels of antioxidant enzymes, including superoxide dismutase 1 (SOD1), superoxide dismutase 2 (SOD2), and catalase, were decreased in CCl_4_- and TAA-treated mice, while the expression levels of these markers were lower in ketogenic diet-fed groups without statistical significance (Figure 6E upper). The detoxification enzymes including Cyp1a2 and Gsta3 were also lower in the CCl_4_- or TAA-alone groups, while the expression levels of Cyp1a2 and Gsta3 were much lower in ketogenic diet-fed groups (Figure 6E lower). These data demonstrated that the ketogenic diet increased the cholesterol quantity in the liver and thereby enhanced liver inflammation and reduced antioxidant and detoxification ability.

## 3. Discussion

A ketogenic diet consists of a high fat content with a moderate protein content and a very low carbohydrate content [9]. It is mainly used in plenty of nervous system diseases like epilepsy and Alzheimer’s disease [21]. It is reported that the ketogenic diet promotes neuroprotective effects by regulating neuronal cell death and oxidative stress [22]. Recently, several studies investigated the function of the ketogenic diet in liver diseases. NAFLD/NASH is one of the risk factors causing liver fibrosis, and approximately 10 to 30% of patients will develop to cirrhosis in the end [23,24,25]. It is reported that losing weight, ameliorating insulin resistance, and reducing hyperlipidemia will help improve NAFLD progression [25]. Studies indicated that the low-carbohydrate ketogenic diet have beneficial effects on patients with NAFLD [26,27,28]. Schugar et al. demonstrated that the ketogenic diet ameliorated NAFLD progression by promoting weight loss and decreasing the amount of triglyceride in the liver [11]. However, they also pointed out that long-term maintenance on a ketogenic diet stimulated the occurrence of NAFLD as well as glucose intolerance [11]. In liver cancer, it is reported that use of a ketogenic diet diminished diethylnitrosamine-induced liver tumor in mice [29]. Previously, we also reported that a ketogenic diet significantly inhibited liver cancer cell growth in NOD/SCID mice [12,13]. Since the influence of the ketogenic diet on liver fibrosis is still unknown, crucial evidence is needed to evaluate the possibility of developing dietary interventions in liver fibrosis. Surprisingly, our data revealed that the ketogenic diet failed to postpone the progression of CCl_4_- or TAA-induced liver fibrosis but aggravated the accumulation of collagen fibers in mice.

To figure out the mechanisms of how the ketogenic diet aggravates fibrosis progression, we further assessed the correlation between ketone bodies and liver fibrosis. Consuming the ketogenic diet can increase the circulatory pool of ketone bodies. bHB and AcAc are the two predominant ketone bodies in the human body [16]. Previous studies showed that treatment with bHB conferred substantial protection against oxidative stress in mice [30]. Chen et al. revealed that bHB has an anti-inflammatory and hepatoprotective role in alcoholic hepatitis [31]. It is also reported that mitochondrial AcAc metabolism in macrophages protects against liver fibrosis [32]. Since the role of both bHB and AcAc in affecting HSC cells activation and liver fibrosis progression remains unclear, we further conducted the in vitro and in vivo experiment in our study. Surprisingly, our data reveal that treatment with bHB and AcAc did not influence hepatic stellate cell activation and the severity of CCl_4_-induced liver fibrosis in mice. Therefore, we know that ketone body production by consuming a ketogenic diet in mice is not the key factor that augments the severity of liver fibrosis. We carefully examined our data again and we found that the H&E staining results demonstrated that the ketogenic diet-fed fibrotic mouse group possessed more lipid accumulation in the liver tissue (Figure 1E and Figure 2E). Moreover, the ketogenic diet increased the cholesterol quantity and further enhanced liver inflammation, and reduced antioxidant and detoxification gene expression in liver. Previous studies had shown that the abnormal metabolism of lipids was prone to inflammation [33]. Besides, the high-fat-diet caused lipid abnormalities, increased oxidative injury, and reduced antioxidant system in rats [34]. Previously, we also reported that higher cholesterol levels in the liver increased the severity of liver fibrosis [19]. Taking these points together, we revealed that the aberrant cholesterol accumulation in liver caused by a ketogenic diet promoted liver inflammation, reduced the antioxidant and detoxification ability, and ultimately aggravated the symptoms of liver fibrosis.

It is important to note that the different composition (percentage of calories from fat, protein, and carbohydrate) of ketogenic diets, as well as the specific genetic background, may exert different metabolic effects between mice and humans. The ketogenic diet has been proposed to be a strategy for reducing obesity. Without carbohydrates in the diet, the ketogenic diet allows the body to induce fatty acid mobilization and promotes weight loss efficiently [35]. Although the ketogenic diet was reported to reduce human obesity and blood pressure, ketogenic diet feeding may increase serum low-density lipoprotein (LDL) and high-density lipoprotein (HDL) significantly [36]. However, some studies indicated that the ketogenic diet could not contribute to weight loss in mice [37], which is consistent with our study (data not shown). Apart from triggering lipid metabolism to generate ketone bodies, gluconeogenesis, the crucial pathway to regulate blood sugar, synthesizes glucose from protein and fat to supplement the lack of carbohydrates in the body [38]. Recent studies indicate that the different protein content of the ketogenic diet may lead to entirely different results. Mice consuming a higher fat to protein ratio may result in body weight gain in comparison with the chow control [38,39]. With a deep study into the different composition of protein in a ketogenic diet, it was found that instead of aiding weight loss, methionine supplementation was more effective than choline in restoring weight gain in mice [40]. Moreover, both methionine and choline did not suppress lipogenic gene expression, but methionine supplementation reversed the upregulated expression of fatty acid oxidation genes [40]. In particular, fibroblast growth factor 21 (FGF21), which has been reported to play a critical role in lipid oxidation [41], was significantly alleviated in methionine supplementation in a ketogenic diet [40]. A previous study showed that hepatic gene expression level and circulating serum concentrations of FGF21 were elevated in ketogenic diet (KD)-fed mice [42]. Besides, refeeding mice after fasting resulted in rapid FGF21 suppression in mice [42]. It is also reported that mice lacking FGF21 failed to respond appropriately to a KD due to impaired ability to mobilize and utilize lipids [42]. These data suggest that FGF21 production and action are important for adaptation under the use of KD to regulate lipid metabolism appropriately. However, a recent study reported that significant differences in FGF21 expression and function were found between mice and humans [43]. Free fatty acid-induced FGF21 expression was found in mice; however, circulating FGF21 concentrations were found to be decreased in humans [44,45]. Besides, humans demonstrated a huge range of FGF21 expression between different individuals. After fasting for 48 h, the FGF21 blood concentrations were not consistent, either showing no effect, modestly increases, or even a drop in FGF21 levels [46,47,48,49]. Mice having an overall higher metabolism in comparison with humans might be one explanation for this discrepancy between mice and humans [43]. Therefore, the role of FGF21 in adaptation to starving for humans is still under debate. As we mentioned above, a KD strongly induced FGF21 in the liver and its circulating levels in mice [42]. In contrast, a KD did not increase FGF21 serum levels in humans [46,48,50]. Moreover, it was also found that ketone bodies appear in the circulation days before FGF21 level induction under long-term fasting [48]. These data further argue against a regulating role for FGF21 in ketogenesis in humans. Therefore, considering the differences in FGF21 expression and physiological functions between mice and humans may help the translation of the experimental findings from bench to bedside. Previous studies revealed that the ketogenic diet reduces blood glucose levels in rested and exercised rats of different ages, with and without pathology [51]. It was also noted that the ketogenic diet possessed advantages, especially for endurance exercise by promoting fat usage for fuel [52]. Monsalves-Alvarez et al. revealed that mice supplemented with bHB increased exercise capacity, with altered in mitochondrial shape that met energetic challenge demands in mouse skeletal muscle [53]. Exercise is an important aspect while using the high-fat ketogenic diet; however, the mice were not trained to exercise in our CCl_4_- and TAA-induced liver fibrosis models. Therefore, severe lipid accumulation and subsequent liver damage occurred. Since a different ketogenic diet composition may exert different metabolic effects, increased sample size and more experimental designs are necessary to clarify the effects of the ketogenic diet on disease treatment.

## 4. Materials and Methods

### 4.1. Mice

Male C57BL/6 mice (7–8 weeks of age) were purchased from Taiwan National Laboratory Animal Center. Mice were fed with a standard chow diet (No. 5001, LabDiet, St. Louis, MO, USA) or a ketogenic diet (5TJQ, TestDiet, composed of 15.6% calories from protein, 82.3% calories from fat, and 2.1% calories from carbohydrates). The fibrosis model was generated using intraperitoneal injection of carbon tetrachloride (CCl_4_)(2 mg/kg, Sigma-Aldrich, St. Louis, MO, USA) or thioacetamide (TAA)(20 mg/kg, Sigma-Aldrich, St. Louis, MO, USA) 2 times weekly for 6 or 10 weeks. The ketone bodies, including DL-β-hydroxybutyric acid (bHB)(300 mg/kg, Sigma-Aldrich, St. Louis, MO, USA) and acetoacetate (AcAc)(200 mg/kg, Sigma-Aldrich, St. Louis, MO, USA), were intraperitoneally injected into mice 6 times weekly for 6 weeks. Each group’s liver tissues and serum were collected at the end of the experiments. All animal procedures were approved by the Institutional Animal Care and Use Committee of Taipei Medical University (LAC-2020-0115, approved May 2020).

### 4.2. Immunohistochemistry Staining and Blood Biochemical Parameters

Liver tissues were fixed with 10% formalin and the sections were stained with hematoxylin and eosin (H&E) for histopathological examination. To assess the extent of fibrosis, Sirius red staining (Abcam, Cambridge, MA, USA) and Masson’s Trichrome staining (Abcam, Cambridge, MA, USA) were carried out following the manufacturer’s instructions. For immunohistochemistry staining of F4/80, liver sections were incubated with antibodies against F4/80 (1:200; Cell Signaling, Beverly, MA, USA) and detected by using the Universal LSABTM2 kit (DakoCytomation, Carpinteria, CA, USA). All sections were investigated by a light microscope (Olympus CKX41, Olympus Corp., Tokyo, Japan). Serum alanine aminotransferase (ALT), cholesterol, and low-density lipoprotein cholesterol (LDLC) were measured with a biochemical analyzer (VetTest, IDEXX, Westbrook, ME, USA).

### 4.3. Cell Culture

LX2 cells and HSC-T6 cells [20] were cultured in Dulbecco’s modified Eagle’s medium (DMEM)(Gibco BRL, Grand Island, NY, USA) with 1% fetal bovine serum (FBS)(HyClone, Logan, UT, USA), penicillin and streptomycin (100 U/mL), nonessential amino acids (0.1 mM), and L-glutamine (2 mM) at 37 °C in a 5% CO_2_ incubator.

### 4.4. TGF-β Treatment

LX2 cells (1 × 10^5^ per well) and HSC-T6 cells (2 × 10^5^ per well) were (1) seeded in 6-well plates and treated with 0, 5, and 10 mM β-hydroxybutyrate (bHB)(Sigma-Aldrich, St. Louis, MO, USA) or acetoacetate (AcAc)(Sigma-Aldrich, St. Louis, MO, USA), and 10 ng/mL TGF-β (R&D Systems, Minneapolis, MN, USA) for 24 h; and (2) seeded in 6-well plates, pretreated with or without 5 and 10 mM bHB or AcAc for 24 h, and further treated with 10 ng/mL TGF-β for 0, 15, 30, and 60 min.

### 4.5. PDGF Treatment

LX2 cells (1 × 10^5^ per well) and HSC-T6 cells (2 × 10^5^ per well) were seeded in 6-well plates, pretreated with or without 5 and 10 mM bHB or AcAc for 24 h, and further treated with 10 ng/mL PDGF (R&D Systems, Minneapolis, MN, USA) for 0, 15, 30, and 60 min.

### 4.6. Cell Viability Assay

LX2 (2 × 10^3^/well) and HSC-T6 (2 × 10^3^/well) cells were seeded in 96-well plates and cultured overnight. The culture medium was replaced with 0, 1, 5, or 10 mM bHB or AcAc, and incubated for 48 h. The culture medium was removed and we added 50 µL of 3-(4,5-dimethylthiazol-2-yl)-2,5-diphenyl tetrazolium bromide (MTT Solution, Sigma-Aldrich, St. Louis, MO, USA) for 2.5 h. Afterwards, 100 µL DMSO was added to each well and incubated for 10 min. Absorbance of the colored solution was measured at optical density (OD) 570 nm with the microplate reader. Untreated cells served as controls. The cell viability was calculated according to the formula: experimental OD value/control OD value × 100%.

### 4.7. Cholesterol Quantification

A commercial Colorimetric Total Cholesterol Quantification Assay Kit (BioVision, Mountain View, CA, USA) was used to analyze total and free cholesterol production. Cholesterol from 5-mg tissue samples was extracted and oxidized by cholesterol oxidase. A commercial probe was added and the result was detected by using spectrophotometry at OD 570 nm.

### 4.8. Western Blotting

Western blotting was performed as previously described [54]. The following antibodies were used in the experiment: anti-α-SMA antibody (1:1000, Abcam, Cambridge, MA, USA), anti-T/p-SMAD2/3 antibody (1:1000, Cell Signaling, Beverly, MA, USA), anti-T/p-MEK antibody (1:1000, Cell Signaling, Beverly, MA, USA), anti-T/p-ERK antibody (1:1000, Cell Signaling, Beverly, MA, USA), anti-T/p-P38 antibody (1:1000, Cell Signaling, Beverly, MA, USA), anti-T/p-JNK antibody (1:1000, Cell Signaling, Beverly, MA, USA), anti-T/p-AKT antibody (1:1000, Cell Signaling, Beverly, MA, USA), and anti-α-tubulin antibody (1:5000, Sigma-Aldrich, St. Louis, MO, USA).

### 4.9. RNA Extraction and Quantitative RT-PCR

TRIzol reagent (Ambion, Carlsbad, CA, USA) was used to extract total RNA. Two micrograms of total RNA was subjected to reverse transcription with High-Capacity cDNA Reverse Transcription Kits (Applied Biosystems, Carlsbad, CA, USA). The relative mRNA level was determined by qPCR with KAPA SYBR FAST qPCR Master Mix (KAPA Biosystems, Boston, MA, USA) and the gene expression was normalized to that of GAPDH. The primers used for qPCR are listed as follows: α-SMA: 5′-GTTCAGTGGTGCCTCTGTCA-3′ (forward, F) and 5′-ACTGGGACGACATGGAAAAG-3′ (reverse, R); collagen type 1 alpha 2 (Col1a2): 5′-TAGGCCATTGTGTATGCAGC-3′ (F) and 5′- ACATGTTCAGCTTTGTGGACC-3′ (R); TGF-β: 5′-CGAAGCGGACTACTATGC-3′ (F) and 5′-GTTGCTCCACACTTGATTT-3′ (R); Desmin: 5′-CAGGCAGCCAATAAGAAC-3′ (F) and 5′-GCCATCTCATCCTTTAGGT-3′ (R); F4/80: 5′-CAAGACTGACAACCAGACG-3′ (F) and 5′-ACAGAAGCAGAGATTATGACC-3′ (R); TNF-α, 5′-TGTAGCCCATGTTGT AGCAAACC-3′ (F) and 5′-GAGGACCTGGGAGTAGATGAGGTA-3′ (R); SOD1: 5′-GCAGGACCTCATTTTAATCCTCACT-3′ (F) and 5′-GTCTCCAACATGCCTCTCTTCAT-3′ (R); SOD2: 5′-CACACATTAACGCGCAGATCA-3′ (F) and 5′-GGTGGCGTTGAGATTGTTCA-3′ (R); Catalase: 5′-GCCTCGCAGAGACCTGATGT-3′ (F) and 5′-CCCCGCGGTCATGATATTAA-3′ (R); Cyp1a2: 5′-TCCTGGACTGACTCCCACAACT-3′ (F) and 5′-GTACTGGGAGAACGCCATCTGTA-3′ (R); Gsta3: 5′-ACAAGATTATCTCGTTGGCAACAG-3′ (F) and 5′-CTTCCACATGGTAGAGGAGTTCAA-3′ (R); GAPDH: 5′-TCACCACCATGGAGAAGGC-3′ (F) and 5′-GCTAAGCAGTTGGTGGTGCA-3′ (R).

### 4.10. Statistic

The in vitro experiment results are expressed as the means ± standard deviation (SD), while the in vivo experiment results are expressed as the means ± standard error of the mean (SEM). The data were analyzed by nonparametric tests in SPSS v20.0 software (SPSS Inc., Chicago, IL, USA). The Mann–Whitney U-test was used to compare 2 independent groups. Differences were considered statistically significant at *p* < 0.05.

## 5. Conclusions

**Figure 7:**
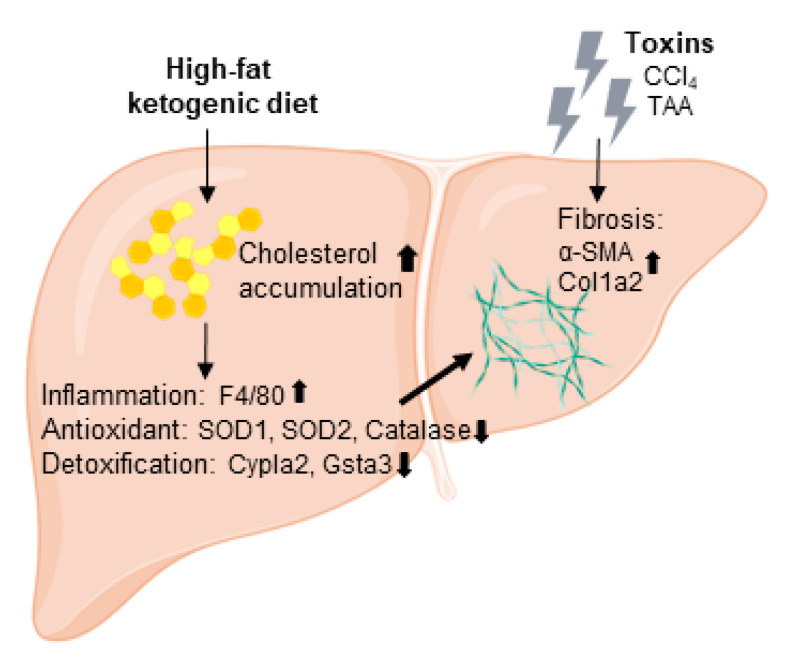
High-fat ketogenic diet feeding may trigger severe steatohepatitis and thereby promote CCl_4_- and TAA- induced liver fibrosis progression through enhanced liver inflammation and the loss of antioxidant and detoxification ability.

Our study provides experimental evidence to support that a high-fat ketogenic diet increased the cholesterol quantity in the liver which further promoted CCl_4_- and TAA- induced fibrotic marker expression, liver inflammation, and the loss of antioxidant and detoxification ability (Figure 7). Furthermore, ketone body (bHB and AcAc) treatment did not influence hepatic stellate cell activation and the severity of liver fibrosis in vivo. Taken together, these results indicate that a high-fat ketogenic diet augments the severity of liver fibrosis in mice.

## Data Availability

Requests for resources and reagents should be contacted to the corresponding author.

## Supplementary Materials

The following are available online at https://www.mdpi.com/1422-0067/22/6/2934/s1. Figure S1: bHB and AcAc supplementation did not influence the proliferation and PDGF-induced phosphorylation of MAPK and AKT pathways in both LX2 and HSC-T6 cells.
