# Supplementary material for: Ketogenic Diet Enhances the Cholesterol Accumulation in Liver and Augments the Severity of CCl4 and TAA-Induced Liver Fibrosis in Mice
Source: Int J Mol Sci. 2021 Mar 13;22(6):2934. doi: 10.3390/ijms22062934 (PMC7998170; doi:10.3390/ijms22062934)
Supplement: Supplementary file 1 [file ijms-22-02934-s001.pdf]

Supplementary Figure 1

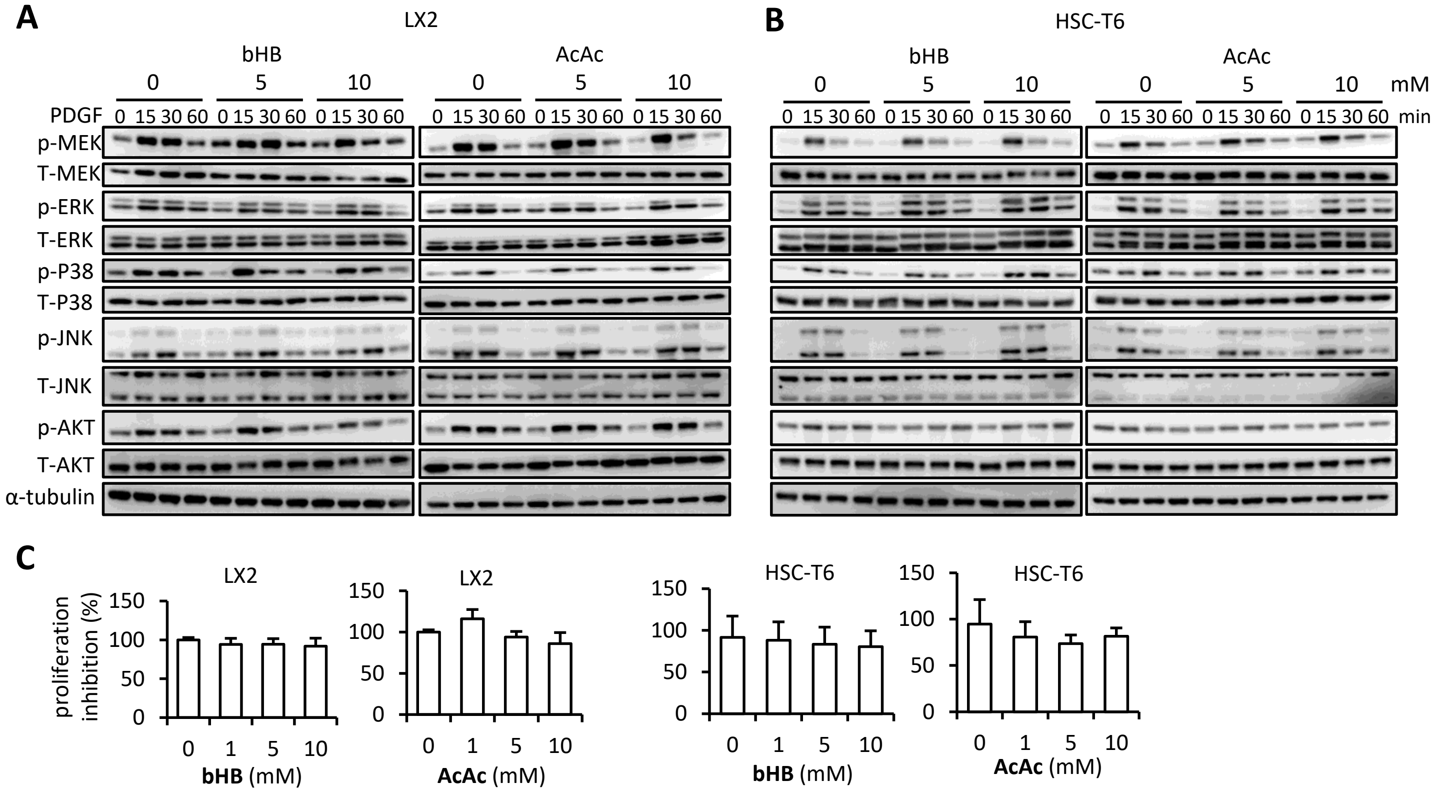

Figure S1. bHB and AcAc supplementation did not influence the proliferation and PDGF-induced phosphorylation of MAPK and Akt pathways in both LX2 and HSC-T6 cells. **(A,B)** LX2 and HSC-T6 cells were pretreated with or without 5, and 10 mM bHB or AcAc for 24 hours and further treated with 10 ng/ml PDGF for 0, 15, 30, and 60 minutes. **(C)** LX2 and HSC-T6 cells were seeded in 96-well plates and incubated with 0, 1, 5, and 10 mM of bHB or AcAc for 48 hours. The cell viability was assessed using MTT assay.
